# Pregnancy and neonatal outcomes in fresh and frozen cycles using blastocysts derived from ovarian stimulation with follitropin delta

**DOI:** 10.1007/s10815-021-02271-5

**Published:** 2021-07-13

**Authors:** Jon Havelock, Anna-Karina Aaris Henningsen, Bernadette Mannaerts, Joan-Carles Arce, ESTHER-1 and ESTHER-2 Trial Groups

**Affiliations:** 1 Pacific Centre for Reproductive Medicine, Department of Obstetrics and Gynaecology, University of British Columbia, Burnaby, BC Canada; 2 Fertility Clinic, Rigshospitalet, Copenhagen University Hospital, Copenhagen, Denmark; 3 Reproductive Medicine & Maternal Health, Ferring Pharmaceuticals, Copenhagen, Denmark

**Keywords:** Follitropin delta, Fresh cycle, Frozen cycle, Neonatal outcome, Ovarian stimulation, Take-home baby rate

## Abstract

**Purpose:**

To describe the pregnancy and neonatal outcomes using fresh and vitrified/warmed blastocysts obtained from ovarian stimulation with follitropin delta in controlled trials versus follitropin alfa.

**Methods:**

This investigation evaluated the outcome from 2719 fresh and frozen cycles performed in 1326 IVF/ICSI patients who could start up to three ovarian stimulations in the ESTHER-1 (NCT01956110) and ESTHER-2 (NCT01956123) trials, covering 1012 fresh cycles and 341 frozen cycles with follitropin delta and 1015 fresh cycles and 351 frozen cycles with follitropin alfa. Of the 1326 first cycle patients, 513 continued to cycle 2 and 188 to cycle 3, and 441 patients started frozen cycles after the fresh cycles. Pregnancy follow-up was continued until 4 weeks after birth.

**Results:**

The overall cumulative take-home baby rate after up to three stimulation cycles was 60.3% with follitropin delta and 60.7% with follitropin alfa (−0.2% [95% CI: −5.4%; 5.0%]), of which the relative contribution was 72.8% from fresh cycles and 27.2% from frozen cycles in each treatment group. Across the fresh cycles, the ongoing implantation rate was 32.1% for follitropin delta and 32.1% for follitropin alfa, while it was 27.6% and 27.8%, respectively, for the frozen cycles. Major congenital anomalies among the live-born neonates up until 4 weeks were reported at an incidence of 1.6% with follitropin delta and 1.8% with follitropin alfa (−0.2% [95% CI: −1.9%; 1.5%]).

**Conclusions:**

Based on comparative trials, the pregnancy and neonatal outcomes from fresh and frozen cycles provide reassuring data on the efficacy and safety of follitropin delta.

**Trial registration:**

ClinicalTrials.gov Identifier: NCT01956110 registered on 8 October 2013; NCT01956123 registered on 8 October 2013.

**Supplementary Information:**

The online version contains supplementary material available at 10.1007/s10815-021-02271-5.

## Introduction

Ovarian stimulation with recombinant follicle-stimulating hormone (FSH) preparations produced using Chinese Hamster Ovary (CHO) cells (i.e., follitropin alfa and follitropin beta) results in acceptable success rates in terms of pregnancy [1] and studies have provided reassuring information regarding the neonatal health after the use of fresh and vitrified/warmed embryos/blastocysts obtained from stimulation with these preparations [2, 3].

Today, recombinant FSH preparations are available from new types of mammalian cell lines. Follitropin delta is the most recently developed recombinant FSH preparation, and it is the first commercially available recombinant FSH expressed from a human cell line (PER.C6®). While follitropin alfa, follitropin beta, and follitropin delta have the same amino acid FSH sequence, follitropin delta resembles native human FSH with α2,6-linked sialic acid and bisecting N-acetylglucosamine, which are not present in follitropin alfa and follitropin beta [4–6]. These characteristics are reflected in a unique pharmacokinetic/pharmacodynamic profile of follitropin delta, resulting in a longer half-life, a slower clearance, and a greater pharmacodynamic response than follitropin alfa [7].

Comprehensive clinical trials have been performed in in vitro fertilization (IVF)/intracytoplasmic sperm injection (ICSI) patients undergoing ovarian stimulation to document the efficacy and safety of follitropin delta versus CHO-derived recombinant FSH preparations [8–13]. The trials have mainly focused on fresh cycles and have provided reassuring information on follitropin delta with respect to the clinical performance of fresh blastocysts leading to pregnancy and live birth as well as the safety of the patients. Nevertheless, two large trials have also included the use of cryopreserved blastocysts in frozen cycles in addition to up to three fresh cycles, facilitating a direct comparison of the cumulative live birth rate per cycle with follitropin delta versus a CHO-derived recombinant FSH preparation [9, 10, 13]. Moreover, neonatal health data have also been collected, including evaluation of congenital anomalies, gestational age, and birth weight. The present integrated analysis provides a comprehensive description of the pregnancy outcomes from women exposed in comparative controlled trials with follitropin delta as well as the neonatal outcomes.

## Materials and methods

### Trial designs

**Fig. 1:**
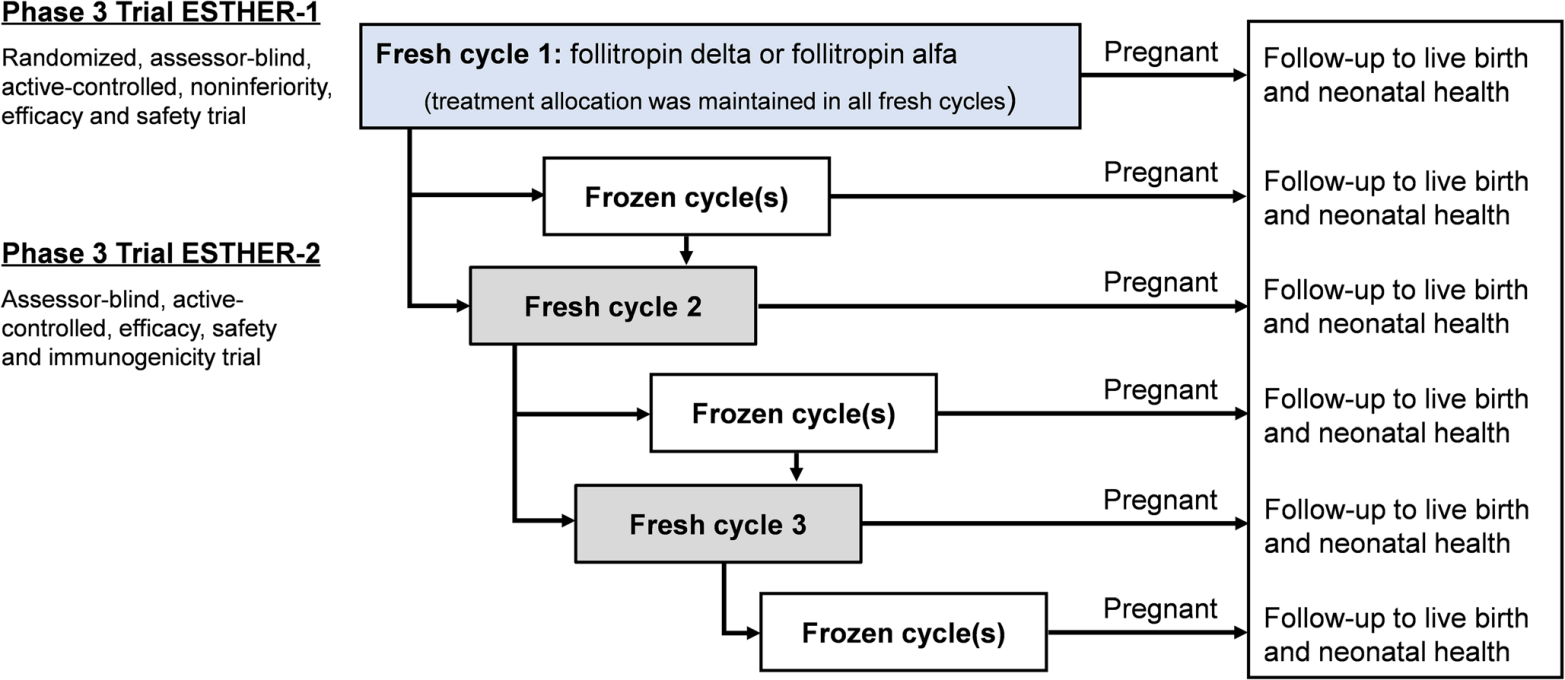
Schematic overview of ESTHER-1 and ESTHER-2 trial designs. The flow of patients is provided for fresh and frozen cycles using day 5 blastocysts obtained after ovarian stimulation with follitropin delta and follitropin alfa. Frozen cycles (optional) were started within 1 year after start of stimulation of the last fresh cycle in either of the trials. *ESTHER* Evidence-based Stimulation Trial with Human rFSH in Europe and Rest of World.

This integrated analysis was conducted with data from two controlled clinical trials in the development program for follitropin delta that were performed between October 2013 and January 2017 at 37 investigational sites in Europe and North and South America. The Evidence-based Stimulation Trial with Human rFSH in Europe and Rest of World 1 (ESTHER-1, NCT01956110) was a randomized, controlled, assessor-blind trial comparing individualized follitropin delta dosing versus conventional follitropin alfa dosing, following a gonadotropin-releasing hormone (GnRH) antagonist protocol (fresh cycle 1) [13]. ESTHER-1 included women who were within the age range 18-40 years, had regular menstrual cycles, and were diagnosed with tubal infertility, unexplained infertility, or endometriosis stage I/II [14], or had a partner diagnosed with male factor infertility. Patients who did not achieve an ongoing pregnancy in ESTHER-1 were offered to participate in the subsequent trial ESTHER-2 (NCT01956123). ESTHER-2 was a controlled, assessor-blind trial and covered up to two additional treatment cycles (fresh cycles 2 and 3) where patients maintained the same treatment allocation to either follitropin delta or follitropin alfa as in the first cycle [9]. Frozen cycles could be performed between or after the fresh cycles as per the patient’s preference (Fig. 1). A detailed patient flow after the first fresh cycle, detailing subsequent frozen cycles and new fresh cycles, has previously been presented [10].

In fresh cycle 1, the follitropin delta (Rekovelle; Ferring Pharmaceuticals, Saint-Prex, Switzerland) dosing regimen was individualized based on patient’s serum anti-Müllerian hormone (AMH, Elecsys® AMH immunoassay; Roche Diagnostics, Rotkreuz, Switzerland) and body weight, and no dose adjustments were made during stimulation. The follitropin alfa (Gonal-f; Merck Serono, Geneva, Switzerland) dose was 150 IU/day for the first 5 days and could thereafter be adjusted. In subsequent cycles, the starting doses were maintained or adjusted according to the ovarian response in the previous cycle. The maximum allowed daily dose of follitropin delta was gradually increased from 12 μg in fresh cycle 1 to 18 μg in fresh cycle 2 and to 24 μg in fresh cycle 3. The maximum allowed daily starting dose of follitropin alfa was 150 IU, 225 IU, and 300 IU in fresh cycles 1, 2, and 3, respectively, with a maximum allowed daily dose of 450 IU in all three cycles. Patients with surplus of day 5 blastocysts as well as patients who underwent triggering of final follicular maturation with GnRH agonist could undergo frozen cycles. Blastocysts were cryopreserved in individual straws using the vitrification method. The transfer policy in the fresh cycles was determined by age and blastocyst quality, with guidance to transfer either one or two blastocysts. In the frozen cycles, one or two blastocysts could be transferred from one or several of the fresh cycles at the discretion of the investigator and patient, and in accordance with local practice. Both natural cycle and programmed regimens were allowed. Pregnancy follow-up was done until 4 weeks after birth for all pregnancies resulting from fresh cycles or frozen cycles started within 1 year after start of stimulation of the last fresh cycle in either of the trials. Additional details on trial design, population and baseline characteristics, procedures, and results, including CONSORT flow diagrams, are available in previous publications [9, 13].

### Outcomes and definitions

The main outcomes were the overall cumulative take-home baby rate for women exposed to follitropin delta and follitropin alfa in the ESTHER trials as well as the neonatal health data up until 4 weeks after birth.

Live birth was defined as the birth of at least one live baby after ≥24 weeks of gestational age. The cumulative take-home baby rate (i.e., live rate at 4 weeks after birth) from fresh and frozen cycles for each stimulation cycle in which the women participated was calculated. A started frozen cycle was defined as a cycle with warming of at least one blastocyst with the intention of transfer. The ongoing implantation rate (defined as the number of intrauterine viable fetuses 10-11 weeks after transfer divided by number of blastocysts transferred) was determined across all fresh cycles as well as all frozen cycles.

Neonatal outcomes included neonatal characteristics such as gestational age, gender, birth weight, and length at birth as well as safety variables like congenital anomalies, stillbirth (defined as death of a fetus after ≥24 weeks of gestation), neonatal death (defined as death of a live-born neonate within 4 weeks after birth), admission to neonatal intensive care unit (NICU) or neonatal care unit (NCU) within 24 h after birth, and also hospitalization occurring between 24 h and 4 weeks after birth.

Singleton and multiple status were based on the number of intrauterine viable fetuses at the ongoing pregnancy visit performed 10-11 weeks after transfer. Gestational age was calculated as the days between blastocyst transfer and birth plus 19 days. A birth weight <2500 g was defined as low birth weight, and <1500 g was defined as very low birth weight [15]. Preterm birth was defined as a live birth at <37 weeks gestation and very preterm birth was defined as <32 weeks gestation [16].

Congenital anomalies detected in fetuses, neonates within 24 h after birth (referred to as “at birth”), and neonates between 24 h and 4 weeks after birth (referred to as “at 4 weeks after birth”) were reported after assessment and diagnosis by the neonate’s physician and coded using the Medical Dictionary for Regulatory Activities (MedDRA) versions 18.1 and 19.1. As per regulatory guidelines, major congenital anomalies were defined as a life-threatening structural anomaly or one likely to cause significant impairment of health or functional capacity and which needs medical or surgical treatment, and minor congenital anomalies were defined as a relatively frequent structural anomaly not likely to cause any medical or cosmetic problems [17]. The categorization of minor and major congenital anomalies was made at the time of database lock.

### Statistical analyses

Descriptive statistics are presented without accounting for multiple records for a patient. Cumulative take-home baby rates were compared between treatments by constructing a two-sided 95% confidence interval (CI) for the estimated mean difference in rates (follitropin delta - follitropin alfa) using the Mantel-Haenszel method to combine risk differences across age strata (<35, 35–37, and 38–40 years). Ongoing implantation rates per started cycle with blastocyst transfer were compared between treatments using a mixed effect logistic regression model with treatment, age stratum, and single/double transfer as fixed factors and patient as random effect, assuming normally distributed log-odds. The estimated mean difference and 95% CI were derived using the delta method. Similarly, the take-home baby rates per started cycle were compared between treatments using a mixed effect logistic regression model with treatment and age stratum as fixed factors and patient as random effect. The 95% CIs were calculated to estimate the mean difference in incidence of major and minor congenital anomalies using the method of Wald. All statistical analyses were performed using the SAS software (SAS Institute Inc., version 9.4, Cary, NC, USA).

## Results

### Pregnancy outcomes

In this integrated analysis of data from comparative controlled trials with fresh and frozen cycles, 1326 women underwent a total of 2719 cycles: 1353 cycles in the follitropin delta group and 1366 cycles in the follitropin alfa group (Table 1). These cycles were distributed as 2027 (74.5%) fresh cycles (1012 cycles in the follitropin delta group and 1015 cycles in the follitropin alfa group) and 692 (25.5%) frozen cycles (341 cycles in the follitropin delta group and 351 cycles in the follitropin alfa group). A total of 1326 IVF/ICSI patients (665 for follitropin delta and 661 for follitropin alfa) were randomized and exposed in fresh cycle 1, of whom 513 patients (252 for follitropin delta and 261 for follitropin alfa) continued to fresh cycle 2 and of these, 188 patients (95 for follitropin delta and 93 for follitropin alfa) also started fresh cycle 3. In total, 441 of the 1326 patients also started frozen cycles (222 for follitropin delta and 219 for follitropin alfa). The average number of started fresh and frozen cycles was 2.0 per patient in the follitropin delta group and 2.1 per patient in the follitropin alfa group, and the average number of cycles with transfer was 1.8 per patient for both treatment groups. Fresh and frozen cycles with transfer of one and two blastocysts are presented in Table S1. After fresh cycle 1, 12.5% (14.1% for follitropin delta and 10.9% for follitropin alfa) of the patients had neither achieved an ongoing pregnancy nor proceeded to further fresh or frozen cycles, while this was the case for 19.7% (17.5% for follitropin delta and 21.8% for follitropin alfa) of the patients after fresh cycle 2.

**Table 1:** Number of started cycles and ongoing implantation rate in fresh and frozen cycles

Outcome	Fresh cycles^a^		Frozen cycles^b^	
	Follitropin delta	Follitropin alfa	Follitropin delta	Follitropin alfa
N	665	661	222	219
Started cycles	1012	1015	341	351
Ongoing implantation rate^c^	312/971 (32.1)	313/976 (32.1)	126/457 (27.6)	132/475 (27.8)

^a^The women underwent a maximum of three fresh cycles

^b^The women underwent a maximum of eight frozen cycles

^c^The number of intrauterine viable fetuses 10–11 weeks after transfer divided by number of blastocysts transferred (%)

*N* total number of women

Across the fresh cycles, the overall ongoing implantation rate per started cycle with transfer was 32.1% for follitropin delta and 32.1% for follitropin alfa (estimated mean difference 0.3% [95% CI: −4.2%; 4.7%]) (Table 1). For the frozen cycles, the overall ongoing implantation rate per started cycle with transfer was 27.6% in the follitropin delta group and 27.8% in the follitropin alfa group (estimated mean difference 0.7% [95% CI: −6.1%; 7.6%]). Thus, comparable ongoing implantation rates were achieved between the two treatment groups after fresh or frozen cycles.

**Fig. 2:**
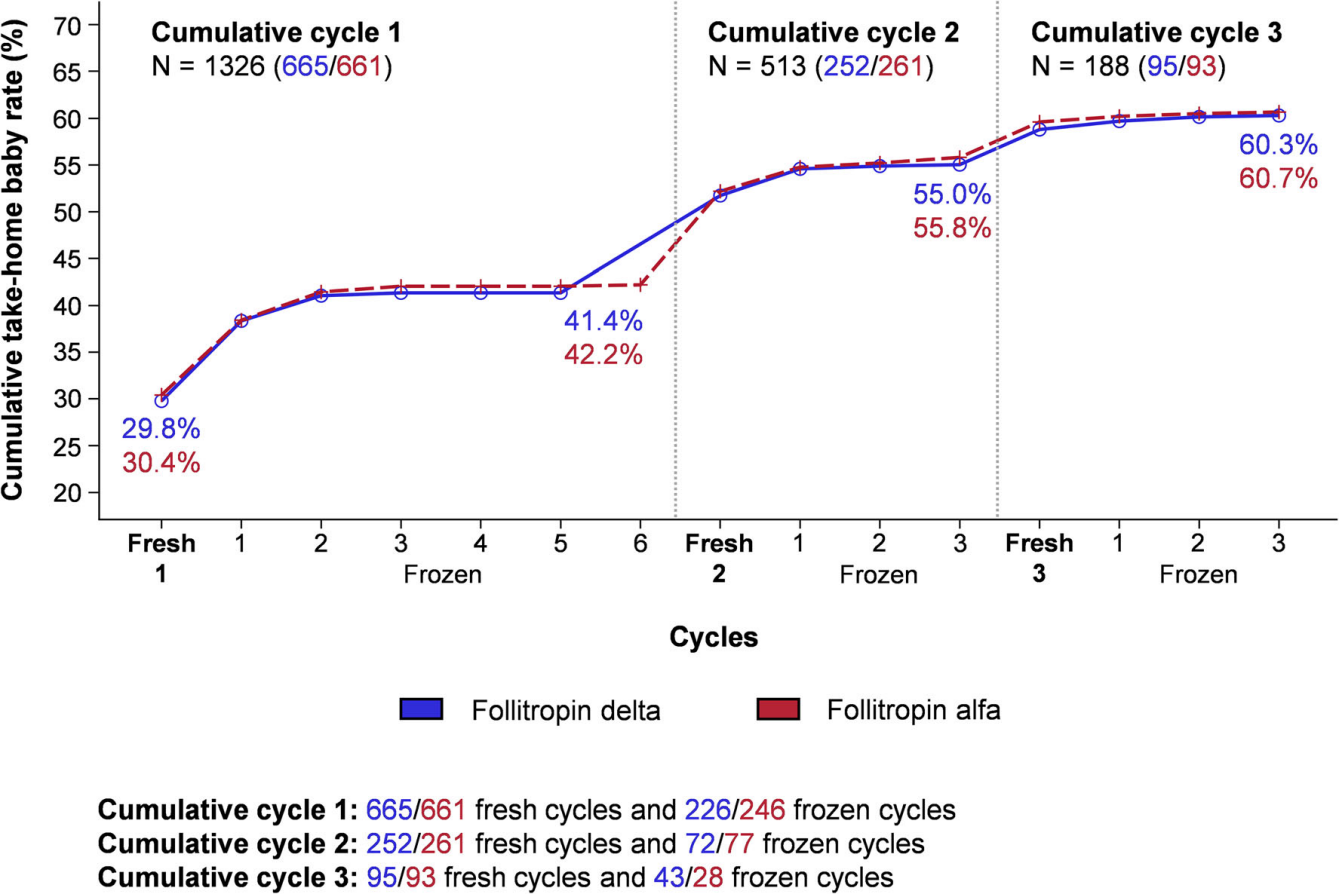
Cumulative take-home baby rate in fresh and frozen cycles. The cumulative take-home baby rate is shown by fresh and frozen cycles with follitropin delta and follitropin alfa. *N* total number of women.

As displayed in Figure 2, the cumulative take-home baby rate after the first ovarian stimulation cycle was 41.4% in the follitropin delta group and 42.2% in the follitropin alfa group. Following the second ovarian stimulation cycle, the cumulative take-home baby rate across the fresh and frozen cycles increased to 55.0% in the follitropin delta group and 55.8% in the follitropin alfa group. The overall take-home baby rate for women who participated in the ESTHER trials was 60.3% with follitropin delta and 60.7% with follitropin alfa (estimated mean difference −0.2% [95% CI: −5.4%; 5.0%]). For the 802 women (401 in each treatment group) with at least one live neonate at 4 weeks after birth, the relative contribution to the take-home baby rate was 72.8% (292/401) from fresh cycles and 27.2% (109/401) from frozen cycles in the follitropin delta group and 72.8% (292/401) from fresh cycles and 27.2% (109/401) from frozen cycles in the follitropin alfa group. The cumulative take-home baby rate is presented by age (<35 and ≥35 years) in Table S2.

In relation to the data obtained in frozen cycles, the availability of cryopreserved blastocysts was similar in the two treatment groups, with 69.5% of the women in the follitropin delta group and 68.8% of the women in the follitropin alfa group having at least one blastocyst cryopreserved (Table S3). The overall survival rate for warmed blastocysts proceeding to transfer was 87.4% in the follitropin delta group and 88.8% in the follitropin alfa group. In each treatment group, 48.1% of the women with frozen blastocysts underwent at least one frozen cycle with transfer within 1 year after start of stimulation of the last fresh cycle. When evaluating the outcome from the frozen cycles, the overall take-home baby rate per started cycle was 32.0% (109/341) in the follitropin delta group and 31.1% (109/351) in the follitropin alfa group (estimated mean difference 1.5% [95% CI: −6.0 %; 8.9 %]).

### Neonatal outcomes

The total number of live-born neonates was 873; distributed as 308 from fresh cycles with follitropin delta, 125 from frozen cycles with follitropin delta, 310 from fresh cycles with follitropin alfa, and 130 from frozen cycles with follitropin alfa (Table S4). In fresh cycles, stillbirth was reported for 2 fetuses in the follitropin delta group and 3 fetuses in the follitropin alfa group. There were no stillbirths in the frozen cycles. Neonatal characteristics of live-born neonates are presented in Table 2 for fresh and frozen cycles. Overall, the mean gestational age for all live-born neonates was 38.7 weeks in both treatment groups, and the birth weight, length at birth, and gender distribution were also similar between treatment groups. The number of singletons and multiples was comparable between the follitropin delta and follitropin alfa groups after fresh and frozen cycles, and there were no higher-order pregnancies than twins. As a result of more double blastocyst transfers in the last fresh cycle and the frozen cycles (Table S1), the number of twins born in these cycles was higher than in the initial fresh cycles; this was observed for both treatment groups. Among singletons and multiples, there was also no difference between treatment groups in neonatal characteristics.

**Table 2:** Characteristics of live-born neonates at birth in fresh and frozen cycles

Characteristic	Fresh cycles		Frozen cycles		Total	
	Follitropin delta	Follitropin alfa	Follitropin delta	Follitropin alfa	Follitropin delta	Follitropin alfa
N	308	310	125	130	433	440
Gestational age (days)						
All	271.0 ± 16.4	271.5 ± 16.1	271.2 ± 14.7	268.6 ± 15.2	271.1 ± 15.9	270.7 ± 15.9
Singletons	273.5 ± 14.3	274.5 ± 11.7	276.5 ± 11.4	274.7 ± 12.1	274.2 ± 13.7	274.6 ± 11.7
Multiples	249.9 ± 18.4	247.5 ± 25.3	256.4 ± 12.5	254.9 ± 12.3	253.2 ± 15.9	251.5 ± 19.6
Gender						
All						
Boy	166 (53.9)	168 (54.2)	58 (46.4)	62 (47.7)	224 (51.7)	230 (52.3)
Girl	142 (46.1)	142 (45.8)	67 (53.6)	68 (52.3)	209 (48.3)	210 (47.7)
Singletons						
Boy	153 (55.4)	149 (54.0)	39 (42.4)	42 (46.7)	192 (52.2)	191 (52.2)
Girl	123 (44.6)	127 (46.0)	53 (57.6)	48 (53.3)	176 (47.8)	175 (47.8)
Multiples						
Boy	13 (40.6)	19 (55.9)	19 (57.6)	20 (50.0)	32 (49.2)	39 (52.7)
Girl	19 (59.4)	15 (44.1)	14 (42.4)	20 (50.0)	33 (50.8)	35 (47.3)
Birth weight (g)						
All	3133 ± 647	3127 ± 610	3160 ± 607	3034 ± 654	3141 ± 635	3100 ± 624
Singletons	3223 ± 586	3229 ± 518	3365 ± 510	3325 ± 491	3258 ± 571	3252 ± 512
Multiples	2359 ± 635	2306 ± 682	2590 ± 482	2377 ± 476	2476 ± 570	2344 ± 577
Length at birth (cm)						
All	49.7 ± 3.6	49.6 ± 3.6	49.9 ± 3.8	49.5 ± 3.9	49.8 ± 3.7	49.6 ± 3.7
Singletons	50.1 ± 3.3	50.0 ± 3.0	50.7 ± 3.6	51.0 ± 3.3	50.3 ± 3.4	50.3 ± 3.1
Multiples	46.3 ± 4.8	46.1 ± 5.8	47.1 ± 2.9	46.0 ± 3.0	46.7 ± 4.0	46.0 ± 4.5

Values are mean ± SD or n (%), unless otherwise stated. Singleton and multiple status were based on the number of intrauterine viable fetuses at the ongoing pregnancy visit

*N* total number of live-born neonates, *n* number of live-born neonates with observations, *SD* standard deviation

As summarized for fresh and frozen cycles in Table 3, the incidence of preterm births was 15.5% in the follitropin delta group and 15.9% in the follitropin alfa group, with a slightly higher incidence in the frozen cycles than in the fresh cycles due to more twin pregnancies. Admission to NICU/NCU immediately after birth, with the most frequent cause being prematurity, and new hospitalization occurring within the initial 4 weeks were reported at a similar incidence in the two treatment groups after fresh and frozen cycles. No neonatal deaths within 4 weeks after birth were reported in the follitropin delta group, while neonatal deaths were reported for 4 neonates in the follitropin alfa group, of which 3 deaths were associated with prematurity and 1 death was caused by a congenital anomaly.

**Table 3:** Prematurity, low birth weight, admission to NICU/NCU, and hospitalization in fresh and frozen cycles

Outcome	Fresh cycles		Frozen cycles		Total	
	Follitropin delta	Follitropin alfa	Follitropin delta	Follitropin alfa	Follitropin delta	Follitropin alfa
N	308	310	125	130	433	440
Preterm birth						
All						
<32 weeks	8 (2.6)	6 (1.9)	1 (0.8)	1 (0.8)	9 (2.1)	7 (1.6)
32–36 weeks	37 (12.0)	35 (11.3)	21 (16.8)	28 (21.5)	58 (13.4)	63 (14.3)
Singletons						
<32 weeks	4 (1.4)	1 (0.4)	1 (1.1)	1 (1.1)	5 (1.4)	2 (0.5)
32–36 weeks	21 (7.6)	18 (6.5)	3 (3.3)	6 (6.7)	24 (6.5)	24 (6.6)
Multiples						
<32 weeks	4 (12.5)	5 (14.7)	0	0	4 (6.2)	5 (6.8)
32–36 weeks	16 (50.0)	17 (50.0)	18 (54.5)	22 (55.0)	34 (52.3)	39 (52.7)
Low birth weight						
All						
<1500 g	8 (2.6)	3 (1.0)	0	1 (0.8)	8 (1.8)	4 (0.9)
1500–2499 g	35 (11.4)	35 (11.3)	14 (11.2)	28 (21.5)	49 (11.3)	63 (14.3)
Singletons						
<1500 g	4 (1.4)	0	0	1 (1.1)	4 (1.1)	1 (0.3)
1500–2499 g	19 (6.9)	19 (6.9)	1 (1.1)	3 (3.3)	20 (5.4)	22 (6.0)
Multiples						
<1500 g	4 (12.5)	3 (8.8)	0	0	4 (6.2)	3 (4.1)
1500–2499 g	16 (50.0)	16 (47.1)	13 (39.4)	25 (62.5)	29 (44.6)	41 (55.4)
Admission to NICU/NCU^a^						
All	29 (9.4)	30 (9.7)	17 (13.6)	22 (16.9)	46 (10.6)	52 (11.8)
Hospitalization^b^						
All	7 (2.3)	12 (3.9)	3 (2.4)	3 (2.3)	10 (2.3)	15 (3.4)

Values are n (%), unless otherwise stated. Singleton and multiple status were based on the number of intrauterine viable fetuses at the ongoing pregnancy visit

^a^Within 24 h after birth

^b^Between 24 h and 4 weeks after birth

*N* total number of live-born neonates, *n* number of live-born neonates with observations, *NCU* neonatal care unit, *NICU* neonatal intensive care unit

### Congenital anomalies

Major congenital anomalies detected among live-born neonates up until 4 weeks after birth are presented in Table 4, with the baseline characteristics for the mothers displayed in Table S5. The incidence of live-born neonates with major congenital anomalies was 1.6% (5/308) in fresh cycles with follitropin delta and 2.3% (7/310) in fresh cycles with follitropin alfa (estimated mean difference −0.6% [95% CI: −2.8%; 1.5%]), while it was 1.6% (2/125) in frozen cycles with follitropin delta and 0.8% (1/130) in frozen cycles with follitropin alfa (estimated mean difference 0.8% [95% CI: −1.8%; 3.5%]). In total, the incidence of live-born neonates with major congenital anomalies was comparable between the two treatment groups, with 1.6% (7/433) for follitropin delta and 1.8% (8/440) for follitropin alfa (estimated mean difference −0.2% [95% CI: −1.9%; 1.5%]). Of these 15 live-born neonates, 10 live-born neonates had only 1 major congenital anomaly, whereas 5 live-born neonates had 2 or 3 major anomalies. The most commonly reported major congenital anomalies were cardiac and vascular disorders as well as renal and urinary tract disorders. In the follitropin delta group, all major congenital anomalies were detected at birth with no new events observed at 4 weeks after birth. Of the 8 live-born neonates with major congenital anomalies in the follitropin alfa group, 5 neonates had major congenital anomalies only at birth, 1 neonate had events both at birth and 4 weeks, and 2 neonates had events only at 4 weeks. The incidence of live-born neonates with major congenital anomalies is presented by patient age (<35 and ≥35 years) in Table S6.

**Table 4:** Major congenital anomalies among live-born neonates in fresh and frozen cycles

MedDRA high-level group term Preferred term	Fresh cycles		Frozen cycles		Total	
	Follitropin delta	Follitropin alfa	Follitropin delta	Follitropin alfa	Follitropin delta	Follitropin alfa
N	308	310	125	130	433	440
Number of neonates with any event (%)	5 (1.6)	7 (2.3)	2 (1.6)	1 (0.8)	7 (1.6)	8 (1.8)
Total number of events	6	11	3	1	9	12
Cardiac and vascular disorders congenital						
Atrial septal defect		1 (0.3)^a^				1 (0.2)^a^
Bicuspid aortic valve		1 (0.3)^a^				1 (0.2)^a^
Coarctation of the aorta		1 (0.3)^a^				1 (0.2)^a^
Double outlet right ventricle				1 (0.8)		1 (0.2)
Patent ductus arteriosus	1 (0.3)		1 (0.8)		2 (0.5)	
Transposition of the great vessels	1 (0.3)				1 (0.2)	
Ventricular septal defect	1 (0.3)	2 (0.6)^a^			1 (0.2)	2 (0.5)^a^
Chromosomal abnormalities and abnormal gene carriers						
Beckwith-Wiedemann syndrome		1 (0.3)^a^	1 (0.8)		1 (0.2)	1 (0.2)^a^
Gastrointestinal tract disorders congenital						
Cleft palate	1 (0.3)	1 (0.3)			1 (0.2)	1 (0.2)
Musculoskeletal and connective tissue disorders congenital						
Adactyly	1 (0.3)				1 (0.2)	
Polydactyly			1 (0.8)		1 (0.2)	
Renal and urinary tract disorders congenital						
Congenital pyelocaliectasis		1 (0.3)				1 (0.2)
Pelvic kidney		1 (0.3)				1 (0.2)
Urethral valves	1 (0.3)	1 (0.3)			1 (0.2)	1 (0.2)
Reproductive tract and breast disorders congenital						
Cryptorchism		1 (0.3)				1 (0.2)

Values are n (%), unless otherwise stated

^a^Detected between 24 h and 4 weeks after birth

*MedDRA* Medical Dictionary for Regulatory Activities, *N* total number of live-born neonates, *n* number of live-born neonates with events

In addition to the major congenital anomalies in live-born neonates, major congenital anomalies leading to elective termination of the pregnancy were reported for 1.1% (5/474) of the clinical pregnancies detected at 5–6 weeks after transfer in the follitropin delta group and 1.0% (5/486) of the clinical pregnancies in the follitropin alfa group after fresh and frozen cycles (estimated mean difference 0% [95% CI: −1.3%; 1.3%]), including 6 cases of trisomy 13, 18, and 21 (4 for follitropin delta and 2 for follitropin alfa). Finally, minor congenital anomalies, defined as relatively frequent structural anomalies not likely to cause any medical or cosmetic problems, were more common than major congenital anomalies. The incidence of live-born neonates with minor congenital anomalies after fresh and frozen cycles was 4.8% (21/433) in the follitropin delta group and 3.0% (13/440) in the follitropin alfa group (estimated mean difference 1.9% [95% CI: −0.7%; 4.5%]).

## Discussion

The present comprehensive data set from comparative controlled trials provided reassurance on all outcomes, including ongoing implantation rate, take-home baby rate, and neonatal health, in fresh and frozen cycles following ovarian stimulation with follitropin delta, a recombinant FSH preparation expressed from a human cell line.

Following follitropin delta treatment, the ongoing implantation rate in fresh cycles, the availability of cryopreserved blastocysts, the survival rate for warmed blastocysts proceeding to transfer, and the ongoing implantation rate in frozen cycles were comparable to follitropin alfa. Moreover, the risk of stillbirth was low, which is in line with previous reports [18]. Across all fresh and frozen cycles, an overall cumulative take-home baby rate of about 60% was achieved in the follitropin delta group. The major relative contribution to the cumulative take-home baby rate was from fresh cycles (approximately three-quarters), and a similar contribution of fresh and frozen cycles was observed within each of the cycles in this analysis. The take-home baby rate reached a plateau after two frozen cycles in the first cumulative cycle and after one frozen cycle in the two next cumulative cycles. In conclusion, these pregnancy outcome findings provide data on the efficacy of follitropin delta in terms of take-home baby rates in fresh and frozen cycles and add reassuring information to the clinical performance of fresh and cryopreserved blastocysts derived from ovarian stimulation with follitropin delta.

In terms of neonatal outcomes, the vast majority of births in these trials resulted in the delivery of healthy neonates. The neonates born after ovarian stimulation with follitropin delta and follitropin alfa demonstrated comparable neonatal outcomes, with no difference regarding gestational age and birth weight. The incidence of congenital anomalies after the use of follitropin delta was within the range of what previous studies on congenital anomalies in neonates born after ovarian stimulation have found [2, 19–25], with the numbers reported in the literature highly dependent on the definitions used and the population studied. The relevant observation, which is made possible by the comparative design of the present data set, is that the overall incidence and distribution of congenital anomalies were similar for the two recombinant FSH preparations in the present analysis. Both major and minor congenital heart anomalies were found after treatment with follitropin delta and follitropin alfa, consistent with this being the most frequently reported anomaly in neonates born after ovarian stimulation [26]. Most importantly, no pattern or clustering of specific types of congenital anomalies was identified.

There are several studies in the literature reporting differences in neonatal outcomes between fresh and frozen cycles [3, 27–33], including higher birth weight in frozen cycles, which was also observed in the present analysis. Although the present data material was too limited to warrant such an analysis, there was no indication of additional concerns regarding neonatal outcomes in either fresh or frozen cycles following ovarian stimulation with follitropin delta compared to follitropin alfa.

The present analysis focused on the take-home baby rate using the data available at 4 weeks after birth. This endpoint is clinically relevant, as it accounts for the losses occurring in the immediate period after birth that are not reflected in the standard reporting of live birth rate. Furthermore, the 4-week follow-up time frame allowed for reporting of those congenital anomalies that are difficult to detect at birth, and comprised 6 of the 21 major congenital anomalies in the present analysis, and therefore also ensured a more complete analysis of neonatal health. The time period of the analyzed frozen cycles covered 1 year after start of the last ovarian stimulation cycle, which seems adequate considering that the cumulative take-home baby rate plateaued after one or two frozen cycles. Nevertheless, this integrated analysis of data was influenced by the individual trial designs, including that a limited proportion of patients proceeded to a new ovarian stimulation cycle before using all cryopreserved blastocysts.

This report compiles the most comprehensive data set of pregnancy and neonatal outcomes in fresh and frozen cycles following ovarian stimulation with a recombinant FSH preparation expressed from a human cell line. The reported take-home baby rate and observed neonatal outcomes with follitropin delta across fresh and frozen cycles in controlled trials with follitropin alfa as a reference add reassuring information on the clinical performance of follitropin delta in terms of efficacy and safety.

### Supplementary information

ESM 1 (DOC 215 kb)

## Data Availability

Not applicable.

## Appendix: The ESTHER-1 and ESTHER-2 Trial Groups’ participating sites and principal investigators are presented in Table 5

**Table 5:** ESTHER-1 and ESTHER-2 Trial Groups’ participating sites and principal investigators

Country	Participating sites and principal investigators^a^
Belgium	Herman Tournaye, UZ Brussel; Petra De Sutter, UZ Gent; Wim Decleer, AZ Jan Palfijn AV, Gent.
Brazil	Alvaro Petracco, Fertilitat – Centro de Medicina Reproductiva, Porto Alegre; Edson Borges, Fertility – Centro de Fertilizacao Assistida, São Paulo; Caio Parente Barbosa, Instituto Ideia Fértil de Saúde Reproductiva, São Paulo.
Canada	Jon Havelock, Pacific Centre for Reproductive Medicine, Burnaby, British Columbia; Paul Claman, Ottawa Fertility Centre, Ottawa, Ontario; Albert Yuzpe, Olive Fertility Centre, Vancouver, British Columbia.
Czech Republic	Hana Visnova, IVF CUBE SE, Prague; Pavel Ventruba, Centre of Assisted Reproduction, Brno; Petr Uher, Institute of Reproductive Medicine and Genetics, Karlovy Vary; Milan Mrazek, GYNEM, Prague.
Denmark	Anders Nyboe Andersen, The Fertility Clinic, Copenhagen University Hospital, Copenhagen; Ulla Breth Knudsen, The Fertility Clinic, Aarhus University Hospital, Skejby.
France	Didier Dewailly, Department of Endocrine Gynaecology and Reproductive Medicine, Hôpital Jeanne de Flandre; Anne Guivarc'h Leveque, Clinique Mutualiste La Sagesse.
Italy	Antonio La Marca, University of Modena and Reggio Emilia, Modena; Enrico Papaleo, Centro Natalità San Raffaele, Milan.
Poland	Waldemar Kuczynski, Kriobank, Bialystok; Katarzyna Kozioł, nOvum Fertility Clinic, Warsaw.
Russia	Margarita Anshina, Centre of Reproduction & Genetics – LLC, Moscow; Irina Zazerskaya, Federal State Budgetary Institution “Federal Center of Heart, Blood & Endocrinology named after V.I. Almazov” of Ministry of Health of the Russian Federation, Saint Petersburg; Alexander Gzgzyan, Institute of Russian Academy of Medical Science Scientific Research Institute of Gynaecology and Obstetrics named after D.O. Ott of North-West Department of RAMS, Saint Petersburg; Elena Bulychova, State Budgetary Health Institution of Moscow Region “Moscow Regional Scientific Research Institute of Obstetrics & Gynaecology.”
Spain	Victoria Verdú, Genefiv, Madrid; Pedro Barri, Hospital Universitario Quirón Dexeus, Barcelona; Juan Antonio García-Velasco, IVI Madrid, Madrid; Manuel Fernández-Sánchez, IVI Sevilla, Seville; Fernando Sánchez Martin, Ginemed, Seville; Ernesto Bosch, IVI Valencia, Valencia; José Serna, IVI Zaragoza, Zaragoza; Gemma Castillon; IVI Barcelona, Barcelona; Rafael Bernabeu, Instituto Bernabeu, Alicante; Marcos Ferrando, IVI Bilbao, Bilbao.
UK	Stuart Lavery, Boston Place Clinic, London, England; Marco Gaudoin, Glasgow Centre for Reproductive Medicine, Glasgow, Scotland.

^a^In total, 37 sites from 11 countries participated in ESTHER-1 and 32 of the 37 sites participated in ESTHER-2

*ESTHER* Evidence-based Stimulation Trial with Human rFSH in Europe and Rest of World
