# Supplementary material for: Pregnancy and neonatal outcomes in fresh and frozen cycles using blastocysts derived from ovarian stimulation with follitropin delta
Source: J Assist Reprod Genet. 2021 Jul 13;38(10):2651–61. doi: 10.1007/s10815-021-02271-5 (PMC8581102; doi:10.1007/s10815-021-02271-5)
Supplement: Supplementary file 1 — (DOC 215 kb) [file 10815_2021_2271_MOESM1_ESM.doc]

**Supplementary Information (SI)**

**Title**

Pregnancy and neonatal outcomes in fresh and frozen cycles using blastocysts derived from ovarian stimulation with follitropin delta

**Journal**

Journal of Assisted Reproduction and Genetics

Authors

Jon Havelock ^1^ • Anna-Karina Aaris Henningsen ^2^ • Bernadette Mannaerts ^3^ • Joan-Carles Arce ^3^ • ESTHER-1 and ESTHER-2 Trial Groups ^4^

**Affiliations of the authors**

^1^ Pacific Centre for Reproductive Medicine, Department of Obstetrics and Gynaecology, University of British Columbia, Burnaby, BC, Canada.

^2^ Fertility Clinic, Rigshospitalet, Copenhagen University Hospital, Copenhagen, Denmark.

^3^ Ferring Pharmaceuticals, Reproductive Medicine & Maternal Health, Copenhagen, Denmark.

^4^ On behalf of the ESTHER-1 and ESTHER-2 Trial Groups, Evidence-based Stimulation Trial with Human rFSH in Europe and Rest of World (see Appendix).

**Corresponding author**

Joan-Carles Arce, jca@ferring.com

**Table S1 Single and double blastocyst transfers** in fresh and frozen cycles

| **Outcome** | **Fresh cycles** | | | | | | | | **Frozen cycles** | | **Total** | |
| --- | --- | --- | --- | --- | --- | --- | --- | --- | --- | --- | --- | --- |
|  | Follitropin delta | | | | Follitropin alfa | | | | Follitropin delta | Follitropin alfa | Follitropin delta | Follitropin alfa |
|  | Cycle 1 | Cycle 2 | Cycle 3 | Total | Cycle 1 | Cycle 2 | Cycle 3 | Total |
|  | |  |  |  |  |  |  |  |  |  |  |  |
| Cycles with transfer | |  |  |  |  |  |  |  |  |  |  |  |
| All | 562 | 211 | 82 | 855 | 560 | 221 | 75 | 856 | 328 | 345 | 1183 | 1201 |
| Single | 539 (95.9) | 168 (79.6) | 32 (39.0) | 739 (86.4) | 536 (95.7) | 171 (77.4) | 29 (38.7) | 736 (86.0) | 199 (60.7) | 215 (62.3) | 938 (79.3) | 951 (79.2) |
| Double | 23 (4.1) | 43 (20.4) | 50 (61.0) | 116 (13.6) | 24 (4.3) | 50 (22.6) | 46 (61.3) | 120 (14.0) | 129 (39.3) | 130 (37.7) | 245 (20.7) | 250 (20.8) |
| Women with transfer | |  |  |  |  |  |  |  |  |  |  |  |
| All | 562 | 211 | 82 | 596 | 560 | 221 | 75 | 602 | 219 | 215 | 614 | 633 |
| Single | 539 (95.9) | 168 (79.6) | 32 (39.0) | 567 (95.1) | 536 (95.7) | 171 (77.4) | 29 (38.7) | 573 (95.2) | 142 (64.8) | 147 (68.4) | 582 (94.8) | 593 (93.7) |
| Double | 23 (4.1) | 43 (20.4) | 50 (61.0) | 94 (15.8) | 24 (4.3) | 50 (22.6) | 46 (61.3) | 99 (16.4) | 105 (47.9) | 102 (47.4) | 180 (29.3) | 188 (29.7) |
|  |  |  |  |  |  |  |  |  |  |  |  |  |
| Values are n (%), unless otherwise stated.  *n* number of cycles/women with transfers. | | | | | | | | | | | | |

**Table S2 Cumulative take-home baby rate in fresh and frozen cycles by patient age**

| **Age** | **Follitropin delta** | | | **Follitropin alfa** | | |
| --- | --- | --- | --- | --- | --- | --- |
|  | n | N | % | n | N | % |
|  |  |  |  |  |  |  |
| <35 years | 258 | 394 | 65.5 | 265 | 392 | 67.6 |
| ≥35 years | 143 | 271 | 52.8 | 136 | 269 | 50.6 |
|  |  |  |  |  |  |  |
| *N* total number of patients, *n* number of patients with observations. | | | | | | |

**Table S3 Blastocyst disposition in frozen cycles**

| **Outcome** | **Fresh cycles** | | | | | | **Total** | |
| --- | --- | --- | --- | --- | --- | --- | --- | --- |
|  | Follitropin delta | | | Follitropin alfa | | | Follitropin delta | Follitropin alfa |
|  | Cycle 1 | Cycle 2 | Cycle 3 | Cycle 1 | Cycle 2 | Cycle 3 |  |  |
| **N** | 665 | 252 | 95 | 661 | 261 | 93 | 665 | 661 |
|  |  |  |  |  |  |  |  |  |
| Number of patients with blastocysts cryopreserved (%) | 402 (60.5) | 129 (51.2) | 34 (35.8) | 402 (60.8) | 107 (41.0) | 31 (33.3) | 462 (69.5) | 455 (68.8) |
| Number of cycles with blastocysts cryopreserved | 402 | 129 | 34 | 402 | 107 | 31 | 565 | 540 |
| Number of blastocysts cryopreserved | 1223 | 355 | 72 | 1392 | 290 | 75 | 1650 | 1757 |
| Number of blastocysts cryopreserved per patient | 1.8 ± 2.4 | 1.4 ± 1.9 | 0.8 ± 1.2 | 2.1 ± 2.6 | 1.1 ± 1.7 | 0.8 ± 1.6 | 2.5 ± 2.8 | 2.7 ± 3.0 |
| Number of blastocysts cryopreserved per patient with at least one blastocyst cryopreserved | 3.0 ± 2.4 | 2.8 ± 1.8 | 2.1 ± 1.1 | 3.5 ± 2.6 | 2.7 ± 1.7 | 2.4 ± 1.9 | 3.6 ± 2.7 | 3.9 ± 2.9 |
| Number of blastocysts warmed | 381 | 122 | 20 | 415 | 97 | 23 | 523 | 535 |
| Number of warmed blastocysts transferred | 342 | 99 | 16 | 372 | 84 | 19 | 457 | 475 |
|  |  |  |  |  |  |  |  |  |
| Values are mean ± SD, unless otherwise stated.  *N* total number of patients, *SD* standard deviation. | | | | | | | | |

**Table S4** Fetus and neonate disposition in fresh and frozen cycles

| **Outcome** | **Fresh cycles** | | | | | | | | **Frozen cycles**^a^ | | **Total** | |
| --- | --- | --- | --- | --- | --- | --- | --- | --- | --- | --- | --- | --- |
|  | Follitropin delta | | | | Follitropin alfa | | | | Follitropin delta | Follitropin alfa | Follitropin delta | Follitropin alfa |
|  | Cycle 1 | Cycle 2 | Cycle 3 | Total | Cycle 1 | Cycle 2 | Cycle 3 | Total |  |  |  |  |
|  |  |  |  |  |  |  |  |  |  |  |  |  |
| Fetuses^b^ | 208 | 75 | 34 | 317 | 217 | 69 | 36 | 322 | 127 | 132 | 444 | 454 |
| Live born neonates |  |  |  |  |  |  |  |  |  |  |  |  |
| All | 201 | 74 | 33 | 308 | 208 | 68 | 34 | 310 | 125 | 130 | 433 | 440 |
| From singleton pregnancy^c^ | 195 | 64 | 17 | 276 | 196 | 64 | 16 | 276 | 92 | 90 | 368 | 366 |
| From multiple pregnancy^c^ | 6 | 10 | 16 | 32 | 12 | 4 | 18 | 34 | 33 | 40 | 65 | 74 |
| Live neonates at 4 weeks after birth |  |  |  |  |  |  |  |  |  |  |  |  |
| All | 201 | 74 | 33 | 308 | 205 | 68 | 34 | 307 | 125 | 129 | 433 | 436 |
| From singleton pregnancy^c^ | 195 | 64 | 17 | 276 | 196 | 64 | 16 | 276 | 92 | 89 | 368 | 365 |
| From multiple pregnancy^c^ | 6 | 10 | 16 | 32 | 9 | 4 | 18 | 31 | 33 | 40 | 65 | 71 |
|  |  |  |  |  |  |  |  |  |  |  |  |  |
| ^a^ The women underwent a maximum of eight frozen cycles.  ^b^ Intrauterine viable fetuses at the ongoing pregnancy visit.  ^c^ Singleton and multiple status were based on the number of intrauterine viable fetuses at the ongoing pregnancy visit. | | | | | | | | | | | | |

**Table S5** Baseline characteristics for patients with a live born neonate with major congenital anomaly

| **Characteristic** | **All patients** | | **Patients with live born neonate with major congenital anomaly** | |
| --- | --- | --- | --- | --- |
|  | Follitropin delta | Follitropin alfa | Follitropin delta | Follitropin alfa |
| **N** | 665 | 661 | 7 | 8 |
|  |  |  |  |  |
| Age (years) | 33.4 ± 3.9 | 33.2 ± 3.9 | 31.4 ± 5.5 | 31.4 ± 3.1 |
| Body weight (kg) | 64.7 ± 10.7 | 63.4 ± 10.4 | 63.5 ± 10.4 | 63.1 ± 8.5 |
| BMI (kg/m^2^) | 23.7 ± 3.4 | 23.3 ± 3.3 | 25.0 ± 4.7 | 23.2 ± 2.5 |
| Antral follicle count | 14.7 ± 6.9 | 14.4 ± 6.8 | 15.0 ± 4.0 | 15.9 ± 10.6 |
| Infertility history |  |  |  |  |
| Duration of infertility (months) | 35.3 ± 24.4 | 34.9 ± 21.7 | 38.9 ± 26.7 | 31.5 ± 16.4 |
| Primary infertility (%) | 70.7 | 71.3 | 85.7 | 62.5 |
| Primary reason for infertility (%) |  |  |  |  |
| Unexplained | 42.3 | 41.3 | 14.3 | 37.5 |
| Tubal | 13.8 | 14.5 | 14.3 | 0 |
| Male factor | 40.3 | 39.3 | 71.4 | 62.5 |
| Endometriosis stage I/II | 3.3 | 4.4 | 0 | 0 |
| Other | 0.3 | 0.5 | 0 | 0 |
|  |  |  |  |  |
| Values are mean ± SD, unless otherwise stated.  *BMI* body mass index, *N* total number of patients, *SD* standard deviation. | | | | |

**Table S6 Live born neonates with major congenital anomalies in fresh and frozen cycles by patient age**

| **Age** | **Follitropin delta** | | | **Follitropin alfa** | | |
| --- | --- | --- | --- | --- | --- | --- |
|  | n | N | % | n | N | % |
|  |  |  |  |  |  |  |
| <35 years | 5 | 280 | 1.8 | 6 | 292 | 2.1 |
| ≥35 years | 2 | 153 | 1.3 | 2 | 148 | 1.4 |
|  |  |  |  |  |  |  |
| *N* total number of live born neonates, *n* number of live born neonates with observations. | | | | | | |
